# Associations between mixtures of urinary phthalate metabolites with gestational age at delivery: a time to event analysis using summative phthalate risk scores

**DOI:** 10.1186/s12940-018-0400-3

**Published:** 2018-06-20

**Authors:** Jonathan Boss, Jingyi Zhai, Max T. Aung, Kelly K. Ferguson, Lauren E. Johns, Thomas F. McElrath, John D. Meeker, Bhramar Mukherjee

**Affiliations:** 1 Department of Biostatistics, University of Michigan School of Public Health, 1415 Washington Heights, Ann Arbor, MI 48109 USA; 2 Department of Environmental Health Sciences, University of Michigan School of Public Health, Ann Arbor, MI USA; 3 Epidemiology Branch, National Institute of Environmental Health Sciences, Research Triangle Park, Durham, NC USA; 4 Division of Maternal and Fetal Medicine, Brigham and Women’s Hospital, Harvard Medical School, Boston, MA USA; 5 Department of Epidemiology, University of Michigan School of Public Health, Ann Arbor, MI USA

**Keywords:** Hazard ratio, Phthalate, Pregnancy, Environmental risk score, Time to delivery, Mixtures

## Abstract

**Background:**

Preterm birth is a significant public health concern and exposure to phthalates has been shown to be associated with an increased odds of preterm birth. Even modest reductions in gestational age at delivery could entail morbid consequences for the neonate and analyzing data with this additional information may be useful. In the present analysis, we consider gestational age at delivery as our outcome of interest and examine associations with multiple phthalates.

**Methods:**

Women were recruited early in pregnancy as part of a prospective, longitudinal birth cohort at the Brigham and Women’s Hospital in Boston, Massachusetts. Urine samples were collected at up to four time points during gestation for urinary phthalate metabolite measurement, and birth outcomes were recorded at delivery. From this population, we selected all 130 cases of preterm birth (< 37 weeks of gestation) as well as 352 random controls. We conducted analysis with both geometric average of the exposure concentrations across the first three visits as well as using repeated measures of the exposure. Two different time to event models were used to examine associations between nine urinary phthalate metabolite concentrations and time to delivery. Two different approaches to constructing a summative phthalate risk score were also considered.

**Results:**

The single-pollutant analysis using a Cox proportional hazards model showed the strongest association with a hazard ratio (HR) of 1.21 (95% confidence interval (CI): 1.09, 1.33) per interquartile range (IQR) change in average log-transformed mono-2-ethyl-5-carboxypentyl phthalate (MECPP) concentration. Using the accelerated failure time model, we observed a 1.19% (95% CI: 0.26, 2.11%) decrease in gestational age in association with an IQR change in average log-transformed MECPP. We next examined associations with an environmental risk score (ERS). The fourth quartile of ERS was significantly associated with a HR of 1.44 (95% CI: 1.19, 1.75) and a reduction of 2.55% (95% CI: 0.76, 4.30%) in time to delivery (in days) compared to the first quartile.

**Conclusions:**

On average, pregnant women with higher urinary metabolite concentrations of individual phthalates have shorter time to delivery. The strength of the observed associations are amplified with the risk scores when compared to individual pollutants.

**Electronic supplementary material:**

The online version of this article (10.1186/s12940-018-0400-3) contains supplementary material, which is available to authorized users.

## Background

Phthalate diesters are produced in large quantities yearly in the US for use in everyday products such as polyvinyl flooring, shower curtains, food packaging plastics, and personal care products. Exposure occurs through contact with these products as well as the consumption of contaminated food and drinking water [1–3]. Phthalate exposure has been related to various health outcomes in humans, including altered thyroid and reproductive hormone levels [4, 5], decreased semen quality in males [6], and asthma and allergic symptoms [7]. Exposure to phthalates in utero has been linked to adverse birth outcomes as well, including altered reproductive tract development in male infants [8], neurodevelopment in both sexes [9, 10], and both prematurity and small size at birth [11–14]. Preterm birth, defined as delivery before 37 weeks completed gestation, is a particularly important endpoint of interest due to: 1) its contribution to neonatal mortality and morbidity and consequent cost to society; 2) the apparent increase in rates over the last three decades; and 3) poorly understood causes and lack of effective interventions [15]. Not only is preterm birth the leading cause of perinatal and infant mortality, but it is also associated with adverse developmental outcomes in children, including chronic conditions such as cardiovascular disease and endocrine disorders [15–17]. The societal costs of preterm birth comprise high medical expenditures and utilization and consequently places economic burdens on healthcare systems [15]. Research to uncover contributing causes, particularly those in connection with environmental contaminant exposures, is a public health priority [18].

We recently demonstrated clear associations between maternal urinary phthalate metabolite concentrations averaged from multiple time points during pregnancy and increased odds of preterm birth in a nested case-control study (*N* = 130 cases, *N* = 352 controls) of women who delivered at the Brigham and Women’s Hospital in Boston [14]. In follow-up analyses we examined variability in phthalate levels across pregnancy, attempted to identify any patterns in levels by gestational age, and assessed associations between phthalate exposure biomarkers at individual time points during pregnancy and preterm birth in order to identify windows of vulnerability [19]. Upon human exposure, phthalates are metabolized quickly, therefore single spot urine samples are less predictive of long-term exposure, and measurement of multiple urine samples are more reliable [20]. While these results suggested that the third trimester of pregnancy may be particularly sensitive for the relationship between phthalate exposure and early delivery, the strongest observed effect estimates were with the more stable metric of average phthalate exposure over gestation.

Studying preterm birth as a binary outcome is reasonable because variation in gestational age at delivery around 40 weeks can be due to misclassification. Thus, examining a cutoff such as 37 weeks focuses on pregnancies that are most likely to be truly early, and that are clinically significant. However, some studies indicate that “preterm birth” is not a homogeneous condition; early delivery—regardless of gestational age—is associated with poorer health outcomes in childhood. For example, late preterm birth (delivery at 34–36 weeks gestation) is associated with increased likelihood of cerebral palsy and other developmental disorders such as reduced mental index scores compared to births that occur at term (after 37 weeks gestation) [21, 22]. Thus, examining the relationship between environmental exposures and the rest of the gestational age distribution could be biologically meaningful.

In this present analysis, we consider time to delivery as our primary outcome of interest instead of the binary outcome of term vs. preterm birth. We consider both average exposure during pregnancy as well as repeated measures of exposure across pregnancy as potential correlates of time to delivery in this framework. In addition to analyzing each compound separately, we constructed two aggregate summaries of total phthalate exposure, an environmental risk score [22] and a weighted quantile sum [23], in relation to time to delivery. These aggregate summary analyses attempt to capture the effect of mixtures on time to delivery.

## Methods

### Study population

Participants were part of an ongoing prospective cohort study of pregnant women with initial prenatal visits at clinics in the Boston area. All women who wished to participate were included if they planned to deliver at the Brigham and Women’s Hospital and if their initial visit was prior to 17 weeks gestation. Subjects were followed throughout the course of pregnancy and provided information (e.g., health status, weight) and urine samples at up to four visits. Urine samples were refrigerated (4 C) for a maximum of two hours before being processed and frozen (− 80 C) for long-term storage. At delivery, birth outcome characteristics such as mode of delivery and fetal measurements were recorded. From 2006 to 2008 approximately 1600 women were recruited, and 1181 were followed until delivery and had live singleton infants. From this population, the present nested case-control study includes all 130 mothers who delivered preterm, as well as 352 controls selected randomly from subjects who had a urine sample from visit 1 and from at least one additional visit.

Gestational ages at individual visits and at delivery were calculated based on last menstrual period (LMP) and confirmed by first trimester ultrasound. Study participants provided written informed consent and institutional review board approval was obtained from Brigham and Women’s Hospital and the University of Michigan. Within this study, visit 1 urine samples were taken at median 9.71 weeks gestation (range 4.71 to 16.1 weeks), visit 2 at median 17.9 weeks (range 14.9 to 21.9 weeks), visit 3 at median 26.0 weeks (range 22.9 to 29.3 weeks), and visit 4 at median 35.1 weeks (range 33.1 to 38.3 weeks). The number of subjects with samples available decreased slightly with increasing visit, with the fourth visit having the smallest number of samples. Visit 4 also had a smaller proportion of cases with urine samples, since some had delivered by this time point.

### Phthalate exposure

Nine phthalate metabolites were measured in each available urine sample (*N* = 1693) by NSF International in Ann Arbor, MI, following methods developed by the Centers for Disease Control (CDC), described in detail elsewhere [24, 25]. The final number of samples analyzed for all phthalate metabolites were as follows by visit (cases, controls): Visit 1 (129, 350); Visit 2 (118, 304); Visit 3 (111, 301); and Visit 4 (66, 314). Phthalate measurements below the limit of detection (LOD) were replaced with the LOD divided by $\sqrt{2}$ [26].

To adjust for urinary dilution, specific gravity (SG) levels were also measured in each urine sample using a digital handheld refractometer (ATAGO Company Ltd., Tokyo, Japan). For univariate analyses phthalate levels were corrected for urinary SG using the following formula: *P*_*C*_ = *P*[(*M*_*SG*_ − 1)/(*SG* − 1)], where *P*_*C*_ represents the SG-corrected phthalate concentration (micrograms per liter), *P* represents the measured concentration in urine, *M*_*SG*_ = 1.015 is the median SG of all samples measured, and *SG* represents the SG of the individual sample [12].

For regression models, unadjusted phthalate levels were used and urinary SG was included as a covariate, since modeling adjusted phthalate levels may incur bias [27]. In the analysis of individual phthalate metabolites, we additionally examined a summed measure of di(2-ethylhexyl) phthalate (DEHP) metabolites (ΣDEHP; nanomoles/liter) that is typically calculated as an index of total exposure to the parent compound. All individual metabolites and ΣDEHP were log-normally distributed and ln-transformed for analysis.

### Descriptive analysis

The nested case-control sample was appropriately weighted via inverse probability weighting in order to compute representative descriptive statistics and to make inference on time to birth for the overall cohort. All analyses utilized these weights. Population-level summary statistics were computed for demographic characteristics, including race, maternal age, education, and health insurance provider (public vs. private). Race, maternal age, and education were included as covariates in all single-pollutant and multi-pollutant analysis models. The distribution of each log-transformed phthalate metabolite and final gestational age were assessed via histogram. To evaluate the concordance between different phthalates, a Pearson correlation matrix between log-transformed and specific gravity corrected average phthalate metabolite concentrations was tabulated.

### Analysis with average exposure

#### Single-pollutant models

One common strategy to model repeated exposure measurements is to compute the average contaminant concentration for each individual and subsequently use the average exposure concentration in the model of interest. In this context, average exposure for a particular phthalate metabolite refers to the average of the log-transformed concentrations obtained at the first three visits. We excluded the fourth visit from our average exposure analysis because women with preterm deliveries were less likely to have a fourth visit. We will consider three such mean exposure analysis models: (1) Logistic Regression, (2) Cox Proportional Hazards Model, and (3) Accelerated Failure Time Model. All coefficients are reported in the unit of one interquartile range (IQR) change of the exposure under consideration. Standardizing by IQR allows us to compare two subjects with identical covariates, one of which is at the 75th percentile of exposure and the other is at the 25th percentile of exposure.

*Logistic Regression with Preterm Birth as Outcome*: Let *T*_*i*_ denote the gestational age at delivery for subject *i* (in days) and let ${E}_i=I\left({T}_i<37\  wks\right)$ indicate whether subject *i* had a preterm birth, where *i* = 1, ⋯, 479. Then the single-pollutant logistic regression model for the *k* th phthalate can be expressed as:

1 $$ \mathit{\log} it\left({\pi}_{ik}\right)={\beta}_{0k}+{\beta}_{1k}{\overline{X}}_{ik}+{\boldsymbol{Z}}_{\boldsymbol{i}}^{\boldsymbol{T}}{\boldsymbol{\beta}}_{\mathbf{2}\boldsymbol{k}}, $$

where ${\pi}_{ik}=P\left({E}_i=1|{\overline{X}}_{ik},{\boldsymbol{Z}}_{\boldsymbol{i}}\right)$, ${\overline{X}}_{ik}$ is the mean of the *k* th phthalate (log-transformed) divided by the IQR of the log-transformed *k* th phthalate for the *i* th individual, *k* = 1⋯, 9, and ${\boldsymbol{Z}}_{\boldsymbol{i}}^{\boldsymbol{T}}$ is a vector of baseline covariates for the *i* th individual (race, education, maternal age, average specific gravity across the first three visits, and health insurance status). The fitted coefficients $\exp \left({\widehat{\beta}}_{1k}\right)$ provide an estimated odds ratio of preterm birth for one IQR increase in average log-transformed phthalate levels. Similar results were presented in Ferguson and colleagues (2014), but are included to compare with the repeated measures single-pollutant exposure results [14].

While logistic regression is easy to implement and interpret, it has the drawback of discretizing gestational age at delivery. Thus, we consider gestational age at delivery as a continuous outcome and fit two commonly used time to event models. The distinction from a standard survival context is that everyone in the study experiences the event and the distribution of time to delivery is left skewed (instead of a survival time distribution, that is typically right skewed).

*Cox Proportional Hazards Model*: The first obvious and natural way to correlate the outcome of gestational age at delivery to phthalate levels is to model the hazard of having birth at time *t, λ*_*k*_(*t*), as a function of the covariates and the *k*-th phthalate metabolite,

2 $$ {\lambda}_k\left(\mathrm{t}\right)={\lambda}_{0k}\left(\mathrm{t}\right)\exp \left({\alpha}_{1k}{\overline{X}}_{ik}+{\boldsymbol{Z}}_{\boldsymbol{i}}^{\boldsymbol{T}}{\boldsymbol{\alpha}}_{\mathbf{2}\boldsymbol{k}}\right), $$

for *k* = 1, ⋯, 9, where ${\overline{X}}_{ik}$ and ${\boldsymbol{Z}}_{\boldsymbol{i}}^{\boldsymbol{T}}$ are defined above. The fitted coefficients $\exp \left({\widehat{\alpha}}_{1k}\right)$ provide an estimated hazard ratio of giving birth at time *t* for a one IQR change in average phthalate exposure (log-transformed). Numbers larger than one indicate an increased hazard ratio of delivery and, therefore, a shorter time to delivery.

*Accelerated Failure Time Model*: Although Cox proportional hazards model is the most commonly used model for time to event data, our direct objective of associating gestational age at delivery (rather than modeling the instantaneous hazard at time *t*) to phthalate levels is better addressed by the accelerated failure time model which, in this case, simply reduces to modeling the log of gestational age as a normal linear regression model:

3 $$ \log \left({T}_i\right)={\gamma}_{0k}+{\gamma}_{1k}{\overline{X}}_k+{Z}_i^T{\gamma}_{2k}+\sigma {\epsilon}_i, $$

where ${\overline{X}}_{ik}$ and ${\boldsymbol{Z}}_{\boldsymbol{i}}^{\boldsymbol{T}}$ are defined above and the errors *ϵ*_*i*_ are independent standard normal variates. Both models provide natural interpretations of their respective parameter estimates. For the accelerated failure time model, $100\times \left(\exp \left({\widehat{\gamma}}_{1k}\right)-1\right)$, yields the percent change in gestational age at delivery per IQR increase in the *k*th log-transformed phthalate exposure. Negative numbers indicate shorter time to delivery.

#### Multi-pollutant models

While the standard practice has been to consider single-pollutant models, in reality, we are exposed to mixtures of multiple agents. A direct approach to modeling multiple pollutants is to construct a joint multivariate model with all 9 phthalate metabolites. This approach is often not feasible due to lack of sample size and potential multicollinearity among exposures. Two recently proposed strategies consider weighted sum type metrics that represent the composite effect of mixtures. The first such method is to construct an environmental risk score (ERS) [22]. ERS is calculated as a linear combination of the individual contaminant exposures, weighted by their associated regression coefficients obtained from a given model. Another method of quantifying aggregate exposure to multiple environmental contaminants is through a weighted quantile sum (WQS), where adaptive weights corresponding to chosen quantiles of phthalates are estimated by bootstrapping the data [23]. In both summative risk scores, the weights are derived from a model of the association between chemical mixtures and the health outcome of interest.

To avoid multicollinearity issues incurred by the inclusion of all 9 phthalates, we adopted two approaches to select subsets of phthalates for ERS and WQS construction. The first approach examined the correlation structure of the phthalates (Additional file 1: Table S1). Five phthalates in the upper-left block show strong correlation (greater than 0.5) and, among them, we chose the one that showed the strongest association with gestational age at delivery (MECPP). The ERS and WQS determined by this empirical examination of correlations were subsequently based on six phthalate metabolites: MECPP, MBzP, MBP, MiBP, MEP, and MCPP. We call the two risk scores based on such ad hoc screening of the correlation structure “ERS-Corr” and “WQS-Corr.” The second approach constituted of running stepwise logistic regression with all 9 candidate phthalates and selecting the phthalates that were retained at the end of the stepwise procedure. The risk scores based on the phthalates selected by stepwise variable selection are called “ERS-Stepwise” and “WQS-Stepwise.” Details regarding data adaptive weight construction for these risk scores are relegated to Additional file 1: Appendix A1.

Analogous to single-pollutant models (1), (2), and (3), similar models with the continuous summative risk scores (or categorized values of the risk scores), generically denoted as *RS* in the following expressions, were fit. All risk scores were standardized by their respective IQR in order to facilitate comparisons between risk scores.

*Logistic Regression*: $logit\left({\pi}_i^{RS}\right)={\beta}_0^{RS}+{\beta}_1^{RS}R{S}_i+{\boldsymbol{Z}}_{\boldsymbol{i}}^{\boldsymbol{T}}{\boldsymbol{\beta}}_{\mathbf{2}}^{\boldsymbol{RS}}$, where ${\pi}_i^{RS}=P\left({E}_i=1|R{S}_i,{\boldsymbol{Z}}_{\boldsymbol{i}}\right)$.

*Cox proportional hazards model*:$\kern0.75em {\lambda}^{RS}(t)={\lambda}_0^{RS}(t)\exp \left({\alpha}_1^{RS}R{S}_i+{\boldsymbol{Z}}_{\boldsymbol{i}}^{\boldsymbol{T}}{\boldsymbol{\alpha}}_{\mathbf{2}}^{\boldsymbol{RS}}\right)$.

*Accelerated failure time model*:$\kern0.75em \log \left({T}_i\right)={\gamma}_0^{RS}+{\gamma}_1^{RS}R{S}_i+{\boldsymbol{Z}}_{\boldsymbol{i}}^{\boldsymbol{T}}{\boldsymbol{\gamma}}_{\mathbf{2}}^{\boldsymbol{RS}}+\sigma {\epsilon}_i$.

### Analysis with repeated measures of exposure

#### Single-pollutant models

In a traditional repeated measures situation, repeated measures are taken on the outcome of interest, potentially adjusted for time independent covariates. However, in our scenario, we have up to four repeated measures per exposure in each subject. To capture the variation in phthalate levels across pregnancy, we consider a two-step method as described in Chen and colleagues (2015) [28]. The two-step method consists of: (a) fitting a linear mixed effects model with random intercepts to the repeated measures of the phthalate levels (b) extracting the estimated subject-specific intercepts to be used as a predictor in the second-step outcome model, akin to the mean analysis presented through models (1), (2), and (3).

Note that the differences between average exposure across visits and subject-specific intercepts are small. However, using subject-specific intercepts is a more general approach as random slopes or other features may be incorporated into the stage 1 linear mixed model (LMM). Moreover, the LMM framework better addresses subjects with differing numbers of visits, because BLUPs are shrinkage estimates of subject-specific averages relative to the population average. In that respect, we feel that the LMM framework is a statistically principled analog to the more ad hoc approach of taking a simple exposure average*.* The details of this fitting process are provided in Additional file 1: Appendix A2.

#### Multi-pollutant models

For the repeated measures analysis, we will only focus on ERS-Corr and ERS-Stepwise (we cannot use WQS, because the outcome in the linear mixed effects model needs to be continuous and WQS is inherently discrete by construction). We repeat the process of constructing the ERS at each time point (Visits 1–4) and fit a random intercept linear mixed model to the repeated measures of ERS at each time point. That is, we first fit:

$$ ER{S}_{ij}={b}_{0i}+{\phi}_0+{\phi}_1{T}_{ij}+{\phi}_2S{G}_{ij}+{\epsilon}_{ij}, $$

where *SG*_*ij*_ is the specific gravity for the *i*th subject at the *j*th visit and ${b}_{0i}\sim N\left(0,{\sigma}_b^2\right)$ and ${\epsilon}_{ij}\sim N\left(0,{\sigma}^2\right)$ are independent. Let ${\widehat{b}}_{0i}$ be the best linear unbiased predictors (BLUP) of the subject-specific random intercepts, extracted from a standard linear mixed effects model output, and let ${\widehat{b}}_{0i}^{\ast }$ denote the IQR standardized BLUP. Then our final analysis models are of the form:

*Logistic regression model*:

$$ logit\left({\pi}_i^{RS}\right)={\beta}_0^{RS}+{\beta}_1^{RS}{\widehat{b}}_{0i}^{\ast }+{\boldsymbol{Z}}_{\boldsymbol{i}}^{\boldsymbol{T}}{\boldsymbol{\beta}}_{\mathbf{2}}^{\boldsymbol{RS}} $$, where $$ {\pi}_i^{RS}=P\left({E}_i=1|{\widehat{b}}_{0i}^{\ast },{\boldsymbol{Z}}_{\boldsymbol{i}}\right) $$.

*Cox proportional hazards model*:

$$ {\lambda}^{RS}(t)={\lambda}_0^{RS}(t)\exp \left({\alpha}_1^{RS}{\widehat{b}}_{0i}^{\ast }+{\boldsymbol{Z}}_{\boldsymbol{i}}^{\boldsymbol{T}}{\boldsymbol{\alpha}}_{\mathbf{2}}^{\boldsymbol{RS}}\right). $$

*Accelerated failure time model*:

$$ \log \left({T}_i\right)={\gamma}_0^{RS}+{\gamma}_1^{RS}{\widehat{b}}_{0i}^{\ast }+{\boldsymbol{Z}}_{\boldsymbol{i}}^{\boldsymbol{T}}{\boldsymbol{\gamma}}_{\mathbf{2}}^{\boldsymbol{RS}}+{\sigma}^{\ast }{\epsilon}_i. $$

All analyses were performed using R statistical software, version 3.4.4 (www.r-project.org). WQS was implemented using the gWQS package in R [29].

## Results

Summary statistics for the study population with respect to demographic characteristics such as race, education, maternal age, and health insurance status can be found in Additional file 1: Table S2. Overall, the cohort primarily had private health insurance and was highly educated, with 79.9% of the study participants having privatized health insurance and 83.6% of women completing some postsecondary education at a college or technical school. There were minimal differences between cases and controls with respect to race, education, maternal age, and health insurance status. Descriptive summary characteristics for the distribution of phthalate metabolite concentrations are provided in supplementary Table S3. All contaminant distributions are right-skewed and each phthalate metabolite has a very low percentage of non-detects, with the largest being 4.70% of values below LOD for MEHP. Additional file 1: Figure S1 shows that the distribution of gestational length is heavily left-skewed.

Table 1 summarizes the odds ratios, hazard ratios, and percent change in the single-pollutant mean exposure analysis models (see Additional file 1: Table S4 for single-pollutant IQR values used in interpreting model coefficients). MEHP (OR: 1.50, 95% CI: 1.10, 2.07), MECPP (OR: 1.66, 95% CI: 1.20, 2.30), and summed DEHP metabolites (OR: 1.47, 95% CI: 1.06, 2.03) all showed elevated odds of preterm birth per IQR change in their respective mean log-transformed concentrations adjusted for average specific gravity, race, education, maternal age, and health insurance status. Considering gestational age as a continuous outcome, the Cox proportional hazards model identifies MECPP (HR: 1.21, 95% CI: 1.09, 1.33), summed DEHP metabolites (HR: 1.14, 95% CI: 1.04, 1.26), MBzP (HR: 1.15, 95% CI: 1.03, 1.27), MBP (HR: 1.17, 95% CI: 1.05, 1.29), and MCPP (HR: 1.10, 95% CI: 1.01, 1.20), as having a significant HR of delivery per IQR change in their respective mean log-transformed concentrations. The single-pollutant accelerated failure time models identify MECPP as having a 1.19% (95% CI: 0.26, 2.11%) decrease in final gestational age in days and summed DEHP as having a 1.03% (95% CI, 0.01, 1.95%) decrease in final gestational age in days for one IQR higher in average log-transformed MECPP and summed DEHP, respectively.

**Table 1:** Single-pollutant associations between average phthalate exposures and gestational age

Phthalate Metabolite	Logistic*		Cox		AFT	
	OR	95% CI	HR	95% CI	% Change	95% CI
MEHP	**1.50**	**(1.10, 2.07)**	1.07	(0.98, 1.17)	−0.79%	(−1.67, 0.09%)
MEHHP	1.05	(0.75, 1.47)	1.04	(0.94, 1.15)	−0.51%	(−1.01, 0.32%)
MEOHP	1.23	(0.87, 1.75)	1.09	(0.98, 1.21)	−0.76%	(−1.74, 0.23%)
MECPP	**1.66**	**(1.20, 2.30)**	**1.21**	**(1.09, 1.33)**	**−1.19%**	**(−2.11, −0.26%)**
Σ DEHP	**1.47**	**(1.06, 2.03)**	**1.14**	**(1.04, 1.26)**	**−1.03%**	**(−1.95, −0.01%)**
MBzP	1.27	(0.79, 1.65)	**1.15**	**(1.03, 1.27)**	−0.60%	(−1.63, 0.43%)
MBP	1.35	(0.99, 1.87)	**1.17**	**(1.05, 1.29)**	−0.74%	(−1.75, 0.28%)
MiBP	0.98	(0.66, 1.45)	1.00	(0.90, 1.12)	0.30%	(−0.82, 1.44%)
MEP	1.22	(0.87, 1.71)	1.01	(0.91, 1.12)	−0.11%	(−1.06, 0.86%)
MCPP	1.27	(0.93, 1.72)	**1.10**	**(1.01, 1.20)**	−0.28%	(−1.18, 0.62%)

Phthalates were averaged across the first three visits and log-transformed. All models were adjusted for average specific gravity, maternal age at first visit, race, and education. The single-pollutant models for MBzP, MBP, MiBP, MEP, and MCPP are also adjusted for health insurance provider. Bolded cells indicate significant (*p* < 0.05) odds ratios (OR), hazard ratios (HR), and percent changes (% Change). Odds ratios, hazard ratios and percent changes are all calculated on IQR (Interquartile Range) scale. Abbreviations: CI, Confidence Interval; Logistic, Logistic Regression; Cox, Cox Proportional Hazards Model; AFT, Accelerated Failure Time Model; MEHP, Mono-(2-ethyl)-hexyl phthalate; MEHHP, Mono-(2-ethyl-5-hydroxyhexyl) phthalate; MEOHP, Mono-(2-ethyl-5-oxohexyl) phthalate; MECPP, Mono-(2-ethyl-5-carboxypentyl) phthalate; Σ DEHP, Summed DEHP metabolites; MBzP, Mono-benzyl phthalate; MBP, Mono-n-butyl phthalate; MiBP, Mono-isobutyl phthalate; MEP, Mono-ethyl phthalate; MCPP, Mono-(3-carboxypropyl) phthalate. *Results are similar to Ferguson and colleagues (2014) [14]

Table 2 summarizes the odds ratios, hazard ratios, and percent change in the single-pollutant repeated measures analysis models using random intercepts (see Additional file 1: Table S4 for BLUP IQR values used in interpreting model coefficients). First-step models adjusted for time-varying specific gravity and all second-step models adjusted for race, education, maternal age, and health insurance status. MEHP (OR: 1.40, 95% CI: 1.06, 1.85), MECPP (OR: 1.43, 95% CI: 1.12, 1.83), and summed DEHP metabolites (OR: 1.32, 95% CI: 1.01, 1.74) showed higher odds of a preterm birth per IQR change in the subject-specific random intercept. MECPP (HR: 1.11, 95% CI: 1.03, 1.19), MBzP (HR: 1.13, 95% CI: 1.05, 1.22), MBP (HR: 1.11, 95% CI: 1.04, 1.19), and MCPP (HR: 1.06, 95% CI: 1.00, 1.12) showed an elevated risk of shortened gestational length per IQR change in their respective mean log-transformed concentrations. Moreover, summed DEHP metabolites had a nearly significant hazard ratio after accounting for the repeated measures of DEHP metabolites (HR: 1.07, 95% CI: 0.99, 1.16). In the accelerated failure time model, MECPP (% Change: -0.74, 95% CI: -1.14, − 0.03%) was the only metabolite that was significantly associated with a percent decrease in final gestational age per IQR change in the subject-specific MECPP random intercept.

**Table 2:** Single-pollutant associations between repeated measures of phthalate exposure and gestational age

Phthalate Metabolite	Logistic		Cox		AFT	
	OR	95% CI	HR	95% CI	% Change	95% CI
MEHP	**1.40**	**(1.06, 1.85)**	1.03	(0.96, 1.12)	−0.52%	(−1.28, 0.26%)
MEHHP	0.97	(0.74, 1.26)	0.98	(0.91, 1.07)	−0.14%	(−0.90, 0.63%)
MEOHP	1.10	(0.85, 1.42)	1.02	(0.95, 1.10)	−0.33%	(−1.07, 0.40%)
MECPP	**1.43**	**(1.12, 1.83)**	**1.11**	**(1.03, 1.19)**	**−0.74%**	**(−1.14, − 0.03%)**
Σ DEHP	**1.32**	**(1.01, 1.74)**	1.07	(0.99, 1.16)	−0.64%	(−1.14, 0.13%)
MBzP	1.08	(0.81, 1.42)	**1.13**	**(1.05, 1.22)**	−0.50%	(−1.28, 0.28%)
MBP	1.18	(0.97, 1.46)	**1.11**	**(1.04, 1.19)**	−0.44%	(−1.11, 0.24%)
MiBP	0.93	(0.72, 1.19)	0.98	(0.92, 1.05)	0.33%	(−0.38, 1.04%)
MEP	1.10	(0.82, 1.46)	0.98	(0.90, 1.06)	0.04%	(−0.78, 0.87%)
MCPP	1.19	(0.95, 1.48)	**1.06**	**(1.00, 1.12)**	−0.19%	(−0.84, 0.46%)

Phthalate measurements at each visit were log-transformed. All models were adjusted for specific gravity at the respective visit, time of sample collection, maternal age at first visit, race, and education. The single-pollutant models for MBzP, MBP, MiBP, MEP, and MCPP are also adjusted for health insurance provider. Bolded cells indicate significant (p < 0.05) odds ratios (OR), hazard ratios (HR), and percent changes (% Change). Odds ratios, hazard ratios and percent changes are all calculated on IQR (Interquartile Range) scale. Abbreviations: CI, Confidence Interval; Logistic, Logistic Regression; Cox, Cox Proportional Hazards Model; AFT, Accelerated Failure Time Model; MEHP, Mono-(2-ethyl)-hexyl phthalate; MEHHP, Mono-(2-ethyl-5-hydroxyhexyl) phthalate; MEOHP, Mono-(2-ethyl-5-oxohexyl) phthalate; MECPP, Mono-(2-ethyl-5-carboxypentyl) phthalate; Σ DEHP, Summed DEHP metabolites; MBzP, Mono-benzyl phthalate; MBP, Mono-n-butyl phthalate; MiBP, Mono-isobutyl phthalate; MEP, Mono-ethyl phthalate; MCPP, Mono-(3-carboxypropyl) phthalate

Overall, results for the repeated measures analysis are consistent with the mean exposure analysis. One notable difference is that the results in Table 2 are generally attenuated relative to the results in Table 1. Intuitively, this is because extracting summaries of phthalates by random effects and associating them with gestational length are “noisier” than directly using averaged measurements.

Descriptive analyses for the phthalate risk scores showed that ERS-Corr and ERS-Stepwise (Additional file 1: Figure S2) are approximately normally distributed, whereas WQS-Corr and WQS-Stepwise are decidedly non-normal. Additional file 1: Figure S3 contains a Pearson correlation matrix between the four risk scores. There are moderately strong, pairwise correlations (average correlation around *r* = 0.6) between ERS-Corr, WQS-Corr, and WQS-Stepwise, but ERS-Stepwise is weakly correlated with WQS-Corr and WQS-Stepwise. Across average exposure and repeated measures models, MEOHP and MECPP had the largest contribution to the construction of ERS-Stepwise and WQS-Stepwise, MECPP, MBP, and MiBP had the largest contribution to the construction of ERS-Corr, and MECPP, MBzP, and MEP had the largest contribution to the construction of WQS-Corr (see Additional file 1: Table S5 for a list of exact weights used in ERS and WQS construction).

Table 3 summarizes the odds ratios, hazard ratios, and percent change in the ERS and WQS average exposure analysis models, where ERS and WQS were determined using mean log-transformed phthalate concentrations. All models were adjusted for specific gravity, race, education, maternal age, and health insurance status. One IQR change in ERS-Corr (OR: 1.81, 95% CI: 1.32, 2.52), ERS-Stepwise (OR: 2.14, 95% CI: 1.62, 2.87), WQS-Corr (OR: 1.66, 95% CI: 1.06, 2.64), and WQS-Stepwise (OR: 1.64, 95% CI: 1.01, 2.72) were all associated with a higher odds of preterm birth. For the Cox proportional hazards model, ERS-Stepwise (HR: 1.30, 95% CI: 1.16, 1.46) and WQS-Corr (HR: 1.21, 95% CI: 1.06, 1.38) showed a significantly higher risk for shortened gestational length per IQR change, while ERS-Corr (HR: 1.06, 95% CI: 0.98, 1.14) and WQS-Stepwise (HR: 1.06, 95% CI: 0.92, 1.23) did not show a significantly higher risk for shortened gestational length per IQR change. In the accelerated failure time model, ERS-Corr (% Change: -1.86, 95% CI: -2.98, − 0.73%), ERS-Stepwise (% Change: -1.84, 95% CI: -2.78, − 0.88%), and WQS-Corr (% Change: -1.12, 95% CI: -2.25, − 0.08%) showed a significant percent decrease in gestational length per IQR change in the respective risk score. See Additional file 1: Table S4 for the IQR ranges of each multi-pollutant risk score.

**Table 3:** Association of gestational age with summative phthalate risk scores

	Logistic		Cox		AFT	
Risk Score	OR	95% CI	HR	95% CI	% Change	95% CI
Average Exposure Analysis						
ERS-Corr	**1.81**	**(1.32, 2.52)**	1.06	(0.98, 1.14)	**−1.86%**	**(−2.98, − 0.73%)**
ERS-Stepwise	**2.14**	**(1.62, 2.87)**	**1.30**	**(1.16, 1.46)**	**−1.84%**	**(−2.78, − 0.88%)**
WQS-Corr	**1.66**	**(1.06, 2.64)**	**1.21**	**(1.06, 1.38)**	**−1.12%**	**(−2.25, −0.08%)**
WQS-Stepwise	**1.64**	**(1.01, 2.72)**	1.06	(0.92, 1.23)	−0.57%	(−1.83, 0.70%)
Repeated Measures Analysis						
ERS-Corr	**1.89**	**(1.45, 2.51)**	**1.19**	**(1.10, 1.27)**	**−1.33%**	**(−2.03, −0.63%)**
ERS-Stepwise	**1.77**	**(1.37, 2.31)**	**1.23**	**(1.14, 1.34)**	**−0.85%**	**(−1.60, − 0.10%)**

Phthalate measurements at each visit were log-transformed. All average exposure models were adjusted for average specific gravity, maternal age at first visit, race, education, and health insurance provider. All repeated measures models were adjusted for specific gravity at each visit, time of sample collection, maternal age at first visit, race, education, and health insurance provider. Bolded cells indicate significant (p < 0.05) odds ratios (OR), hazard ratios (HR), and percent changes (% Change). Odds ratios, hazard ratios and percent changes are all calculated on IQR (Interquartile Range) scale. Abbreviations: CI, Confidence Interval; Logistic, Logistic Regression; Cox, Cox Proportional Hazards Model; AFT, Accelerated Failure Time Model

**Fig. 1:**
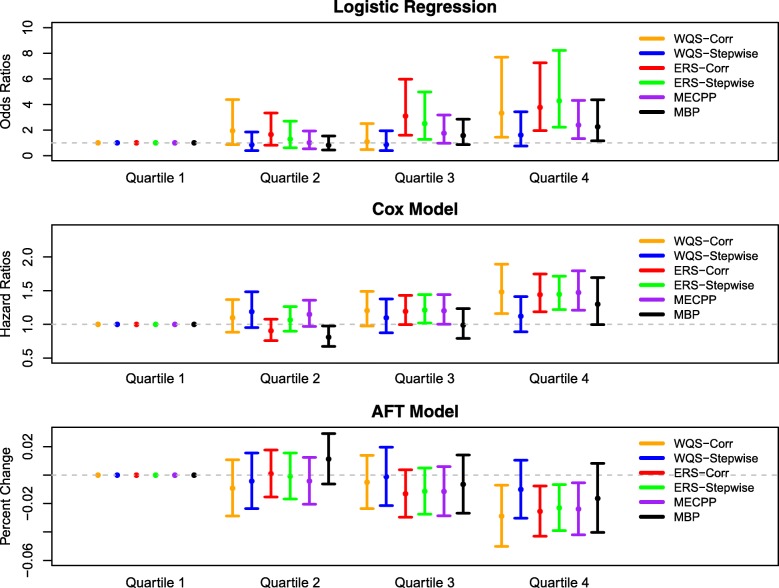
Forest plot of associations between gestational age and risk score quartiles (reference category is quartile 1). ERS/WQS was generated from the average exposure analysis and categorized into quartiles. Single-pollutant average exposure models for MECPP and MBP, where MECPP and MBP are split into quartiles, are also included. Models were adjusted for average specific gravity, maternal age at first visit, race, education, and health insurance provider. Exact numerical results can be found in Additional file 1: Table S6

Figure 1 depicts the odds ratios, hazard ratios, and percent change in gestational age for ERS and WQS quartiles (see Additional file 1: Table S6 for numerical summaries). For WQS-Corr, ERS-Corr, and ERS-Stepwise, there is generally an increasing trend in the odds ratios and hazard ratios and a decreasing trend in the % change as the respective risk score quartile increases. Namely, WQS-Corr shows significantly higher odds of preterm birth (OR: 3.33, 95% CI: 1.44, 7.69), significantly higher risk for shortened gestational length (HR: 1.48, 95% CI: 1.16, 1.89), and a significant percent decrease in gestational length (% Change: -2.89, 95% CI: -5.01, − 0.71%) for quartile 4 compared to quartile 1.

Two single-pollutant mean exposure models for MECPP and MBP are also included in Fig. 1 for comparison. Notably, the odds ratios, hazard ratios, and % change corresponding to the single-pollutant models are attenuated relative to the ERS-Corr, ERS-Stepwise, and WQS-Corr models. Specifically, if we compare quartile 4 to quartile 1, then we see that ERS-Corr has an odds ratio of 3.77 (95% CI: 1.96, 7.25), a hazard ratio of 1.44 (95% CI: 1.19, 1.75), and a % change of − 2.55% (95% CI: -4.30, − 0.76%), while the single-pollutant mean exposure models for MBP have an odds ratio of 2.25 (95% CI: 1.16, 4.36), a hazard ratio of 1.30 (95% CI: 1.00, 1.69), and % change of − 1.63% (95% CI: -4.03, 0.82%). Thus, measures of aggregate phthalate exposure, notably ERS-Corr, ERS-Stepwise, and WQS-Stepwise, quantify a stronger association between phthalate exposure and gestational length, compared to single-pollutant models.

Table 3 also summarizes the odds ratios, hazard ratios, and percent change in the ERS exposure analysis models, where ERS is determined using repeated measures of phthalate concentrations. For ERS-Corr (OR: 1.89, 95% CI: 1.45, 2.51; HR: 1.19, 95% CI: 1.10, 1.27; % Change: -1.33, 95% CI: -2.03, − 0.63%) and ERS-Stepwise (OR: 1.77, 95% CI: 1.37, 2.31; HR: 1.23, 95% CI: 1.14, 1.34; % Change: -0.85, 95% CI: -1.60, − 0.10%) we observe an elevated odds, higher risk of lower gestational length, and a percent decrease in gestational length per IQR change in their respective ERS (see Random Intercept column in Additional file 1: Table S4 for repeated measures ERS IQR ranges).

## Discussion

In this paper, we make two primary contributions. The first is to analyze gestational age at delivery in a time to event framework. Modeling time to delivery as a continuous variable addresses the limitations of using dichotomous outcomes such as term versus preterm birth, which may oversimplify the pathological consequences of shorter gestational periods. The second is to use phthalate risk scores, such as ERS and WQS, as summary measures to estimate the cumulative effect of phthalate mixtures. We examined four different risk scores, and found that three of the four, ERS-Corr, ERS-Stepwise, and WQS-Corr, were significantly associated with time to delivery. Simulation studies need to be conducted to better understand the analytical benefits and drawbacks of using ERS compared to WQS.

Overall, this study provides further evidence that select phthalates are associated with risk factors for adverse reproductive and birth outcomes. Namely, several individual phthalates, such as MECPP and summed DEHP metabolites, were associated with an increased odds of preterm delivery, an increased risk of delivering, and a reduced gestational duration. Associations from the repeated measures analysis for individual pollutants appear to be attenuated compared to the associations from the average exposure analysis. This is primarily due to the limited number of repeated measures for each subject and the additional variability that is introduced as a result of computing the BLUP estimates in the first-stage model. When comparing the models with multi-pollutant risk scores to single pollutant models, we found that ERS-Corr and WQS-Corr were associated with an even greater reduction in gestational duration and higher odds of preterm birth in comparison to individual phthalate metabolites. These findings align with the hypothesis that mixtures of multiple pollutants may have greater adverse effects in comparison to single-pollutants evaluated in isolation.

Analytically, logistic regression, Cox proportional hazards model, and accelerated failure time model (AFT) estimate different quantities, and thus the significance and interpretation of single phthalate metabolites and summative phthalate risk scores are likely to vary across the three models. Logistic regression estimates odds ratios, intrinsically related to a dichotomized outcome and most commonly used measure in this context. The hazard ratio estimate obtained from the Cox proportional hazards model can be understood as a ratio of the hazard rates for women with high and low phthalate exposure, respectively. A hazard ratio greater than one indicates that a woman with greater phthalate exposure has a higher probability of instantaneously giving birth compared to a woman with lower phthalate exposure, given that both women have not delivered up to that time point. Although the Cox proportional hazards model is very popular for time to event data, the interpretation in the context of gestational duration is somewhat unusual as everybody experiences the event and the time to delivery data is left skewed instead of being right skewed (typically noted for survival outcomes). The AFT model has a much simpler interpretation; namely, a negative coefficient implies that, on average, women with higher phthalate exposure would expect a reduction is gestational duration compared to women with lower phthalate exposure. Given the ease of interpretation on the direct gestational age scale, we believe that AFT is better suited for studies of time to gestation.

Preterm delivery, defined as gestational duration less than 37 weeks, can be an informative and clinically relevant outcome to predict maternal and child health outcomes. However, there are also other dichotomous cutoffs, such as late preterm (34–36 weeks gestation), moderate preterm (32–33 weeks gestation), very preterm (< 32 weeks gestation) and extremely premature birth (< 28 weeks gestation) [30, 31]. Even among term pregnancies that result in delivery after 37 weeks gestation, there is considerable variation in days of gestation prior to delivery [32]. Furthermore, post-term births that take place after 42 weeks gestation also contribute to perinatal morbidity [30]. There may be distinct and overlapping pathological consequences associated with each of these gestational age ranges, therefore, analyzing continuous gestational age as an outcome variable may characterize a more accurate understanding of the relationship between maternal phthalate levels and overall duration of pregnancy. Our study reported a reduction in gestational duration that ranged between 0 and 3% for most predictor variables, which we recognize may not be clinically significant on the individual level. However, given the ubiquity of phthalate exposure, we emphasize that the reduction in gestational duration associated with phthalate exposure, averaged among all births that take place in the U.S. population, could have widespread societal level effects.

Several investigators have previously characterized associations between concentrations of phthalate metabolites during pregnancy and either gestational age or preterm birth, however, their methodological approach differed from our present study – previous studies have mostly estimated associations with gestational age through linear regression [12, 13, 31, 33–39]. Although these previous studies do not address phthalate mixtures, some of these studies have found parallel findings to our single-pollutant analyses. Weinberger and colleagues (2013) assessed the relationship between phthalate metabolites and gestational age in 72 women from New Jersey, and reported that maternal urinary MEHHP was associated with a decrease in gestational age [31]. In another study of 68 women from Michigan, Watkins and colleagues (2016) observed an inverse relationship between the sum of DBP metabolites (MBP, MHBP, MCPP) at delivery and gestational age [38]. Polanska and colleagues (2016) observed significant inverse associations between maternal MEP in the third trimester and gestational age from a prospective birth cohort in Poland (*N* = 165) [36]. In a cohort of strictly African American and Dominican women in New York (*N* = 331), Whyatt and colleagues (2009) found that shorter gestational duration was significantly associated with higher maternal urinary concentrations of MEHP, MEHHP, MEOHP, and MECPP in the third trimester [13]. In addition to these studies, a small case-control study of women from Mexico (*N* = 60) reported that maternal urinary MECPP, MBP, and MCPP during the third trimester was significantly associated with an increase in the odds of preterm birth [12].

Aside from maternal urinary levels of phthalate metabolites, one of the studies focused on metabolite concentrations in cord blood [34]. In this study of 207 women from China, associations between cord blood levels of phthalates and gestational age were estimated [34]. Concentrations of several phthalates in cord blood (DMP, DEP, DEEP, DPP, BMPP, DNHP, BBP, DNOP, DMEP, DBP, DIBP, DBEP, and DNP) were significantly associated with shorter gestational age [34]. Another study in Italy (*N* = 84) found lower gestational age among infants with detectable cord blood concentrations of MEHP in comparison to infants without detectable MEHP [35].

Among the existing studies reviewed here, some have also found contrary or null results in comparison to our study. Adibi and colleagues (2009) drew from a multicenter U.S. pregnancy cohort (*N* = 283), and found maternal urinary concentrations of the metabolites MEHP, MEOPP, and MEHPP to be significantly associated with lower odds of preterm birth [33]. Meanwhile, these investigators also reported significant increase in odds for delivery past 41 weeks gestation in relation to higher urinary concentrations of MEHP, MEOHP, and MEHPP [33]. Similar to these findings, a study of 404 women in New York also reported a positive association between maternal MEHP concentrations in the third trimester in relation to longer gestational age [39]. Another study of pregnant women in Japan (*N* = 149) by Suzuki and colleagues (2010) resulted in non-significant associations between 9 different phthalate metabolites (MMP, MEP, MnBP, MBzP, MEHP, MEHHP, and MEOHP) and gestational age [37]. Contrasting findings from previous studies may be due to differences in exposure assessment – most of these studies measured phthalates using single spot urine samples. Another reason could be due to geographical dissimilarities in the location of study participants, given that phthalate exposure may vary by country and region.

Phthalates are metabolized quickly in the body, and as a mixture, they may be interacting with several target tissues to elicit changes in various endogenous signaling molecules, such as hormones, and markers of inflammation and oxidative stress [20, 40]. There are several potential mechanisms by which phthalate mixtures can disrupt the production and circulation of endogenous biomarkers, due to their ability to interact with nuclear receptors and transcription factors, such as estrogen and progesterone receptors, aryl hydrocarbon receptors, peroxisome proliferator-activated receptors, and thyroid receptors [40–42]. Human and animal studies indicate that select phthalate metabolites have been associated with disruption of several circulating hormones, inflammation and oxidative stress markers [42–45]. With concern for reproductive health, phthalate exposure may alter gestational duration by acting through these mechanistic pathways. Through shortened gestational duration, phthalate exposure may contribute to adverse neonatal outcomes and child morbidity later in life.

Though we present a comprehensive analytical framework to capture time and multiple pollutants in an omnibus analysis, there are several methodological limitations. First, the accelerated failure time model in our analysis, which is typically used for right-skewed outcomes, is modeling a left-skewed outcome, namely gestational duration. Therefore, as a sensitivity check, we fit accelerated failure time models on a transformed version of gestational age, such that the log of the transformed gestational age was normally distributed (results not presented). In terms of significance and direction, transformed and untransformed time to delivery produced consistent findings. Regression parameters for the models with untransformed time to delivery are more straightforward to interpret, however, in modeling a log-transformed left-skewed outcome, estimates of the percent reduction in gestational age may be heavily influenced by a small number of subjects with very short gestational duration. Second, ERS is calculated and used on the same data and thus has the potential for overfitting. Before using the phthalate ERS as a prognostic tool, one needs to validate it in an independent cohort. Third, we did not collect data on dietary patterns prior to or during pregnancy, which may confound the relationship between phthalates and gestational duration.

One major challenge in multipollutant modeling is the selection of etiologically relevant contaminant mixtures in the presence of potentially highly collinear exposures. WQS is specifically designed to handle moderately correlated predictors, however variable selection properties of WQS under a high degree of multicollinearity are not well-studied. Czarnota and colleagues (2015) argued that variable selection in the presence of moderate multicollinearity using an ad hoc threshold for WQS weights outperforms regularized regression methods such as elastic net, but there is no theoretical justification for their claim or the choice of the threshold [46]. Moreover, Czarnota and colleagues (2015) note that they expect WQS to have worse performance when subject to highly correlated contaminants [46]. Given that logistic regression is known to have poor performance under strong multicollinearity, we would also expect our ERS construction method to have difficulty with highly collinear phthalate metabolites [47]. In such situations, ridge regression type methods may have more desirable properties for constructing risk scores though they do not lead to unbiased estimates of each of the separate coefficients.

In our multipollutant models, ERS is based on a model with linear phthalate main effects and does not capture potential interactions or non-linearity in the response surface. However, there are multiple strategies for constructing exposure risk scores that do simultaneously account for nonlinearity and high order interactions in the response surface [48]. Bayesian Additive Regression Trees (BART) sum individual regression trees together to estimate a flexible multivariable function of exposures that is associated with the health outcome of interest [49]. Similar to BART, Bayesian Kernel Machine Regression (BKMR) also aims to estimate a multivariable function of exposures that is associated with the health outcome of interest, but instead uses kernels to approximate a wide array of possible functional forms [50]. Both methods should be considered when it is contextually important to incorporate interactions between exposures into the ERS generative model.

Additionally, we want to point out that ERS and WQS are both typically calculated using the same structure of the analysis model. Ideally, the ERS should be constructed based on fitting Cox regression on training data and validating on the test data. However, in our present analysis, ERS and WQS are each generated from a logistic regression model and are subsequently used as explanatory variables in Cox proportional hazards models and AFT models. The main issue with using continuous gestational age in ERS/WQS construction is that ERS/WQS needs to be generated separately for Cox regression and AFT, because model parameters for Cox regression and AFT correspond to different interpretable quantities (hazard ratio and percent reduction in gestational age, respectively) and are on different scales. Another reason for using ERS from models with preterm birth as an outcome is that most of the published data are available on this outcome rather than considering gestational age as a time to event outcome. Thus if other investigators wanted to construct ERS based on coefficients reported in other published studies, the ERS we proposed would be comparable.

Lastly, our study is also limited by the reality that phthalates are highly variable, and measurements reflect recent exposures [20]. We previously reported interclass correlation coefficients (ICC) of phthalates from this study population [19], which represents the ratio of intra-individual variability to the sum of intra and inter-individual variability and range from zero to one, where values equal to one indicate no intra-individual variability [51]. ICC for phthalates in this study population ranged from 0.19 to 0.61, indicating low to moderate intra-individual variability. As such, our exposure assessment of phthalates may suffer from some degree of non-differential measurement error.

Despite these limitations, our study has several strengths. First, we obtained up to four urine samples from a large cohort of pregnant women. Compared to single spot urine measurements, having multiple repeated measurements affords a robust exposure assessment, and reduces non-differential measurement error due to intra-individual phthalate variability. Our study also used highly sensitive analytical methods to measure urinary phthalate metabolites, which resulted in high detection rates. Finally, our study was strengthened by our assessment of gestational dates, which were validated both clinically and with first-trimester ultrasonography.

Finally, we would like to emphasize that for non-detects below the LOD, substitution by LOD/$\sqrt{2}$ may not always lead to optimal statistical properties. This is less of a concern in our analysis as a very small proportion of phthalate metabolite concentrations are below their respective LODs (Table S3). For studies with higher proportions of non-detects one may want to use more rigorous approaches for handling non-detects, such as multiple imputation or censored likelihood maximization [52, 53]. In such situations, LOD/$\sqrt{2}$ substitution could heavily bias regression parameter estimates for constructing ERSs, even if the proportion of non-detects is relatively small, i.e., around 15–20% [54–56].

## Conclusions

Environmental exposure to phthalates remain a persistent public health concern, especially within the context of pregnancy. The present study determined that several phthalates and phthalate risk scores, which quantify the isolated effect of a single phthalates and the aggregate effect of multiple phthalates, respectively, were associated with shortened gestational duration in the Cox proportional hazards models, the accelerated failure time models, and logistic regression models. Furthermore, this study provides a novel statistical framework for investigators to analyze the simultaneous effect of multiple pollutants. Future studies should aim to characterize potential biological mediators that relate phthalate exposure and gestational duration.

## Additional file

Additional file 1: Outlining descriptive characteristics of the nested case-control study population, ERS and WQS Construction, and additional modeling details. **Table S1.** Pearson correlation between mean sg-adjusted phthalate exposures. **Table S2.** Descriptive statistics for pregnant women in the nested case-control sample. **Table S3.** Descriptive Statistics for Phthalate Metabolites. **Table S4.** Interquartile Range (IQR) Standardization Values. **Table S5.** Weights for ERS and WQS Construction. **Table S6.** Association between preterm birth / gestational age and categorized phthalate risk score. **Figure S1.** Distribution of final gestational age for subjects included in the nested case-control sample. **Figure S2.** Distribution of ERS and WQS corresponding to the average exposure analysis. **Figure S3.** Scatterplot matrix and Pearson correlation between phthalate risk scores. Appendix A1: Construction of ERS and WQS in Average Exposure Analysis. Appendix A2: Single-pollutant models with repeated measures of exposure. (DOCX 469 kb)

## Abbreviations

BLUP: Best linear unbiased predictors
DEHP: Di(2-ethylhexyl) phthalate
ERS: Environmental risk score
IQR: Interquartile range
LMP: Last menstrual period
LOD: Limit of detection
MBP: Mono-n-butyl phthalate
MBzP: Mono-benzyl phthalate
MCPP: Mono-(3-carboxypropyl) phthalate
MECPP: Mono-(2-ethyl-5-carboxypentyl) phthalate
MEHHP: Mono-(2-ethyl-5-hydroxyhexyl) phthalate
MEHP: Mono-(2-ethyl)-hexyl phthalate
MEOHP: Mono-(2-ethyl-5-oxohexyl) phthalate
MEP: Mono-ethyl phthalate
MiBP: Mono-isobutyl phthalate
SG: Specific gravity
WQS: Weighted quantile sum
