# Supplementary material for: Associations between mixtures of urinary phthalate metabolites with gestational age at delivery: a time to event analysis using summative phthalate risk scores
Source: Environ Health. 2018 Jun 20;17:56. doi: 10.1186/s12940-018-0400-3 (PMC6011420; doi:10.1186/s12940-018-0400-3)
Supplement: Supplementary file 1 — Outlining descriptive characteristics of the nested case-control study population, ERS and WQS Construction, and additional modeling details. Table S1. Pearson correlation between mean sg-adjusted phthalate exposures. Table S2. Descriptive statistics for pregnant women in the nested case-control sample. Table S3. Descriptive Statistics for Phthalate Metabolites. Table S4. Interquartile Range (IQR) Standardization Values. Table S5. Weights for ERS and WQS Construction. Table S6. Association between preterm birth / gestational age and categorized phthalate risk score. Figure S1. Distribution of final gestational age for subjects included in the nested case-control sample. Figure S2. Distribution of ERS and WQS corresponding to the average exposure analysis. Figure S3. Scatterplot matrix and Pearson correlation between phthalate risk scores. Appendix A1: Construction of ERS and WQS in Average Exposure Analysis. Appendix A2: Single-pollutant models with repeated measures of exposure. (DOCX 469 kb) [file 12940_2018_400_MOESM1_ESM.docx]

**Table S1** Pearson correlation between mean sg-adjusted phthalate exposures

|  | MEHP | MEHHP | MEOHP | MECPP | ∑DEHP | MBZP | MBP | MiBP | MEP | MCPP |
| --- | --- | --- | --- | --- | --- | --- | --- | --- | --- | --- |
| MEHP | **1.00** | **0.70** | **0.78** | **0.71** | **0.83** | 0.06 | 0.07 | 0.12 | -0.01 | 0.32 |
| MEHHP | **0.70** | **1.00** | **0.92** | **0.67** | **0.84** | 0.07 | 0.12 | 0.13 | -0.04 | 0.33 |
| MEOHP | **0.78** | **0.92** | **1.00** | **0.88** | **0.97** | 0.09 | 0.16 | 0.19 | 0.01 | 0.40 |
| MECPP | **0.71** | **0.67** | **0.88** | **1.00** | **0.95** | 0.07 | 0.12 | 0.19 | 0.06 | 0.40 |
| ∑DEHP | **0.83** | **0.84** | **0.97** | **0.95** | **1.00** | 0.07 | 0.13 | 0.18 | 0.02 | 0.41 |
| MBZP | 0.06 | 0.07 | 0.09 | 0.07 | 0.07 | **1.00** | 0.47 | 0.40 | 0.20 | 0.18 |
| MBP | 0.07 | 0.12 | 0.16 | 0.12 | 0.13 | 0.47 | **1.00** | 0.42 | 0.26 | 0.43 |
| MiBP | 0.12 | 0.13 | 0.19 | 0.19 | 0.18 | 0.40 | 0.42 | **1.00** | 0.26 | 0.20 |
| MEP | -0.01 | -0.04 | 0.01 | 0.06 | 0.02 | 0.20 | 0.26 | 0.26 | **1.00** | 0.12 |
| MCPP | 0.32 | 0.33 | 0.40 | 0.40 | 0.41 | 0.18 | 0.43 | 0.20 | 0.12 | **1.00** |

Bolded entries in the Pearson correlation matrix indicate correlations above 0.5.

**Table S2** Descriptive statistics for pregnant women in the nested case-control sample

|  | Median (25th-75th percentiles) / N (%) | | |
| --- | --- | --- | --- |
| Demographic Characteristics | Overall  (N=482) | Cases  (N=130) | Controls  (N=352) |
| Maternal age | 32.7 (29.0-35.7) | 32.8 (29.3-35.8) | 32.7 (28.7-35.7) |
| Race/ethnicity |  |  |  |
| White | 282 (58.5) | 75 (57.7) | 207 (58.8) |
| African American | 77 (16.0) | 22 (16.9) | 55 (15.6) |
| Other | 123 (25.5) | 33 (25.4) | 90 (25.6) |
| Education |  |  |  |
| High school | 68 (14.1) | 21 (16.2) | 47 (13.4) |
| Technical school | 77 (16.0) | 25 (19.2) | 52 (14.8) |
| Junior college or some college | 139 (28.8) | 38 (29.2) | 101 (28.7) |
| College graduate | 187 (38.8) | 45 (34.6) | 142 (40.3) |
| Missing | 11 (2.3) | 1 (0.8) | 10 (2.8) |
| Health insurance |  |  |  |
| Private insurance/HMO/self-pay | 385 (79.9) | 108 (83.1) | 277 (78.7) |
| Medicaid/SSI/MassHealth | 85 (17.6) | 20 (15.4) | 65 (18.5) |
| Missing | 12 (2.5) | 2 (1.5) | 10 (2.8) |

Summary statistics are weighted by the probability of inclusion for each subject in the nested case-control to generalize demographic characteristic summary statistics to the overall birth cohort.

**Table S3** Descriptive Statistics for Phthalate Metabolites

|  |  |  |  | Percentile | | | | |
| --- | --- | --- | --- | --- | --- | --- | --- | --- |
| Phthalate | LOD | % < LOD | GM (GSD) | 25 | 50 | 75 | 90 | Max |
| MEHP | 1.00 | 4.70% | 10.98 (3.48) | 4.76 | 9.43 | 21.83 | 56.63 | 1555.26 |
| MEHHP | 0.10 | 0.00% | 34.09 (3.44) | 14.76 | 27.82 | 70.91 | 187.11 | 2850.00 |
| MEOHP | 0.10 | 0.10% | 18.47 (3.35) | 8.40 | 15.54 | 38.31 | 93.75 | 1128.00 |
| MECPP | 0.20 | 0.00% | 45.34 (3.43) | 18.36 | 37.93 | 101.59 | 248.87 | 3712.50 |
| ∑DEHP | NA | NA | 0.40 (3.19) | 0.17 | 0.32 | 0.81 | 2.03 | 21.10 |
| MBzP | 0.20 | 1.10% | 7.13 (3.05) | 3.45 | 6.48 | 13.41 | 29.15 | 465.00 |
| MBP | 0.50 | 0.30% | 18.30 (2.58) | 10.84 | 16.68 | 28.12 | 47.91 | 24879.31 |
| MiBP | 0.10 | 0.40% | 7.62 (2.29) | 4.75 | 7.61 | 12.02 | 19.47 | 350.62 |
| MEP | 1.00 | 0.10% | 143.49 (4.71) | 47.46 | 124.62 | 393.41 | 1111.18 | 48130.43 |
| MCPP | 0.20 | 3.20% | 2.16 (3.20) | 1.05 | 1.72 | 3.55 | 8.92 | 847.83 |

Unweighted summary statistics and percent below the detection limit (LOD) for each phthalate metabolite. Phthalate Metabolites are reported in μg/L and ∑DEHP is reported in μmol/L. Abbreviations: GM, Geometric Mean; GSD, Geometric Standard Deviation.

| **Table S4** Interquartile Range (IQR) Standardization Values | | | |
| --- | --- | --- | --- |
| Single-pollutant IQRs | | | |
| Phthalate Metabolite | Abbreviation | Mean | Random Intercept |
| Mono-(2-ethyl)-hexyl phthalate | MEHP | 1.39 | 0.68 |
| Mono-(2-ethyl-5-hydroxyhexyl) phthalate | MEHHP | 1.44 | 0.51 |
| Mono-(2-ethyl-5-oxohexyl) phthalate | MEOHP | 1.46 | 0.43 |
| Mono-(2-ethyl-5-carboxypentyl) phthalate | MECPP | 1.50 | 0.63 |
| Summed DEHP metabolites | Σ DEHP | 1.34 | 0.50 |
| Mono-benzyl phthalate | MBzP | 1.56 | 0.94 |
| Mono-n-butyl phthalate | MBP | 1.25 | 0.62 |
| Mono-isobutyl phthalate | MiBP | 1.25 | 0.58 |
| Mono-ethyl phthalate | MEP | 1.94 | 1.20 |
| Mono-(3-carboxypropyl) phthalate | MCPP | 1.37 | 0.57 |
| Multi-pollutant Risk Score IQRs | | | |
| Risk Score | Abbreviation | Mean | Random Intercept |
| ERS Correlation | ERS-Corr | 0.58 | 0.11 |
| ERS Stepwise | ERS-Stepwise | 0.74 | 0.11 |
| WQS Correlation | WQS-Corr | 1.10 | NA |
| WQS Stepwise | WQS-Stepwise | 1.68 | NA |

Table S4 provides a list of IQR values that were used to standardize metabolite of risk scores prior to model fitting in order to facilitate better model interpretation. The column “Mean” corresponds to mean log-adjusted phthalate metabolites or risk scores constructed from mean phthalate metabolites, while the “Random Intercept” column corresponds to the IQR of the BLUPs used in the repeated measures analysis models.

| **Table S5** Weights for ERS and WQS Construction | | | | | | |
| --- | --- | --- | --- | --- | --- | --- |
|  | Mean | | Visit 1 | Visit 2 | Visit 3 | Visit 4 |
| Phthalate | ERS | WQS | ERS | ERS | ERS | ERS |
| Selection Via Phthalate Correlation Matrix | | | | | | |
| MECPP | 0.36 | 0.33 | 0.20 | 0.09 | 0.22 | 0.09 |
| MBzP | 0.02 | 0.21 | -0.08 | -0.10 | 0.12 | -0.06 |
| MBP | 0.26 | 0.04 | 0.18 | 0.20 | 0.17 | 0.50 |
| MiBP | -0.27 | 0.01 | -0.12 | -0.02 | -0.23 | -0.50 |
| MEP | 0.07 | 0.33 | 0.07 | 0.08 | 0.03 | 0.01 |
| MCPP | -0.07 | 0.08 | 0.07 | -0.13 | 0.00 | 0.00 |
| Stepwise Selection | | | | | | |
| MEHP | 0.42 | 0.33 | 0.10 | 0.54 | 0.12 | 0.49 |
| MEHHP | 0.76 | 0.06 | 0.61 | 0.21 | 0.42 | 0.88 |
| MEOHP | -2.29 | 0.33 | -1.72 | -1.45 | -1.37 | -3.03 |
| MECPP | 1.36 | 0.16 | 1.21 | 0.73 | 0.99 | 1.69 |
| MBP | 0.32 | 0.09 | 0.32 | 0.23 | 0.30 | 0.55 |
| MiBP | -0.31 | 0.02 | -0.08 | -0.02 | -0.13 | -0.30 |

Weights under the objective selection header correspond to ERS-Corr and WQS-Corr, while the weights under the stepwise selection header refer to ERS-Stepwise and WQS-Stepwise. The “Mean” header refers to the ERS and WQS generate from the average exposure analysis and Visit X refers to the ERS generated from measurements taken at Visit X. The logistic regression model that generates ERS is adjusted for average specific gravity/specific gravity at each visit, maternal age at first visit, race, education, and health insurance provider. The logistic regression model that generates WQS is adjusted for maternal age at first visit, race, education, and health insurance provider.

| **Table S6** Association between preterm birth / gestational age and categorized phthalate risk score | | | | | | | |
| --- | --- | --- | --- | --- | --- | --- | --- |
|  |  | Logistic | | Cox | | AFT | |
| Risk Score | Quartile | OR | 95% CI | HR | 95% CI | % Change | 95% CI |
| WQS-Corr | 2 | 1.95 | (0.87, 4.38) | 1.10 | (0.88, 1.37) | -0.92% | (-2.88%, 1.08%) |
|  | 3 | 1.08 | (0.47, 2.50) | 1.20 | (0.97, 1.49) | -0.50% | (-2.35%, 1.39%) |
|  | 4 | **3.33** | **(1.44, 7.69)** | **1.48** | **(1.16, 1.89)** | **-2.89%** | **(-5.01%, -0.71%)** |
| WQS-Stepwise | 2 | 0.85 | (0.39, 1.85) | 1.19 | (0.95, 1.48) | -0.42% | (-2.36%, 1.55%) |
|  | 3 | 0.87 | (0.39, 1.94) | 1.10 | (0.88, 1.38) | -0.11% | (-2.15%, 1.97%) |
|  | 4 | 1.61 | (0.76, 3.42) | 1.12 | (0.89, 1.41) | -1.01% | (-3.03%, 1.06%) |
| ERS-Corr | 2 | 1.65 | (0.82, 3.33) | 0.90 | (0.76, 1.08) | 0.10% | (-1.54%, 1.76%) |
|  | 3 | **3.09** | **(1.60, 5.97)** | 1.19 | (1.00, 1.43) | -1.31% | (-2.96%, 0.37%) |
|  | 4 | **3.77** | **(1.96, 7.25)** | **1.44** | **(1.19, 1.75)** | **-2.55%** | **(-4.30%, -0.76%)** |
| ERS-Stepwise | 2 | 1.29 | (0.62, 2.69) | 1.07 | (0.90, 1.26) | -0.08% | (-1.69%, 1.56%) |
|  | 3 | **2.51** | **(1.26, 4.97)** | **1.21** | **(1.02, 1.44)** | -1.14% | (-2.75%, 0.50%) |
|  | 4 | **4.28** | **(2.23, 8.22)** | **1.45** | **(1.22, 1.72)** | **-2.30%** | **(-3.91%, -0.66%)** |
| MECPP | 2 | 1.02 | (0.54, 1.92) | 1.15 | (0.97, 1.36) | -0.42% | (-2.05%, 1.24%) |
|  | 3 | 1.75 | (0.97, 3.18) | **1.20** | **(1.00, 1.44)** | -1.15% | (-2.86%, 0.59%) |
|  | 4 | **2.39** | **(1.32, 4.31)** | **1.47** | **(1.21, 1.79)** | **-2.39%** | **(-4.20%, -0.54%)** |
| MBP | 2 | 0.82 | (0.44, 1.53) | **0.81** | **(0.67, 0.97)** | 1.13% | (-0.63%, 2.92%) |
|  | 3 | 1.57 | (0.86, 2.85) | 0.99 | (0.79, 1.23) | -0.65% | (-2.67%, 1.42%) |
|  | 4 | **2.25** | **(1.16, 4.36)** | **1.30** | **(1.00, 1.69)** | -1.63% | (-4.03%, 0.82%) |

Table S6 provides a summary of the multi-pollutant models where the ERS/WQS generated from the average exposure analysis is categorized into quartiles, and subsequently used as explanatory variables in each model (reference category is quartile 1). Analogous single-pollutant average exposure models for MECPP and MBP, where MECPP and MBP are split into quartiles are also included. Models were adjusted for average specific gravity, maternal age at first visit, race, education, and health insurance provider. Bolded cells indicate significant (p<0.05) odds ratios (OR), hazard ratios (HR), and percent changes (% Change). Odds ratios, hazard ratios and percent changes are all calculated on IQR (Interquartile Range) scale. Abbreviations: CI, Confidence Interval; Logistic, Logistic Regression; Cox, Cox Proportional Hazards Model; AFT, Accelerated Failure Time Model.


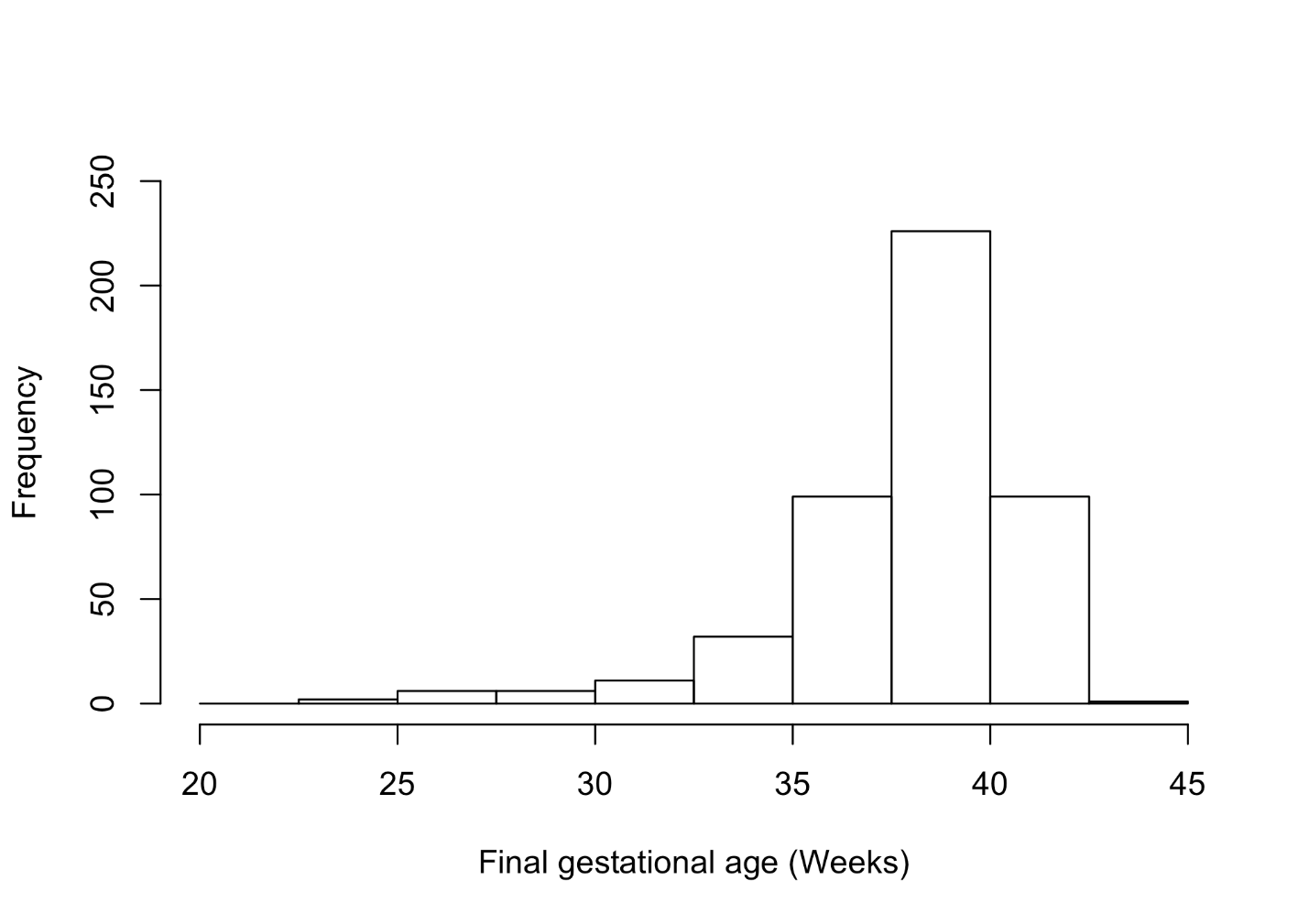


**Fig. S1** Distribution of final gestational age for subjects included in the nested case-control sample.


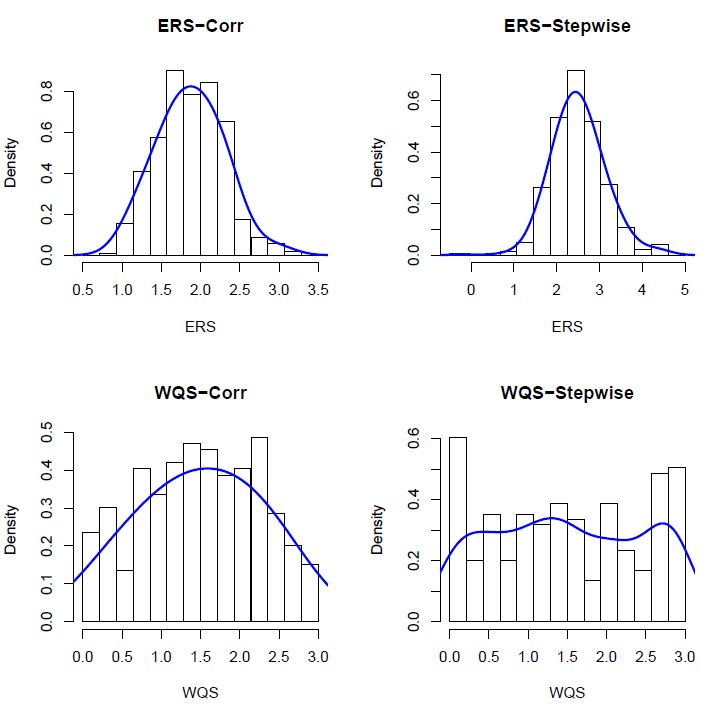


**Fig. S2** Distribution of ERS and WQS corresponding to the average exposure analysis.


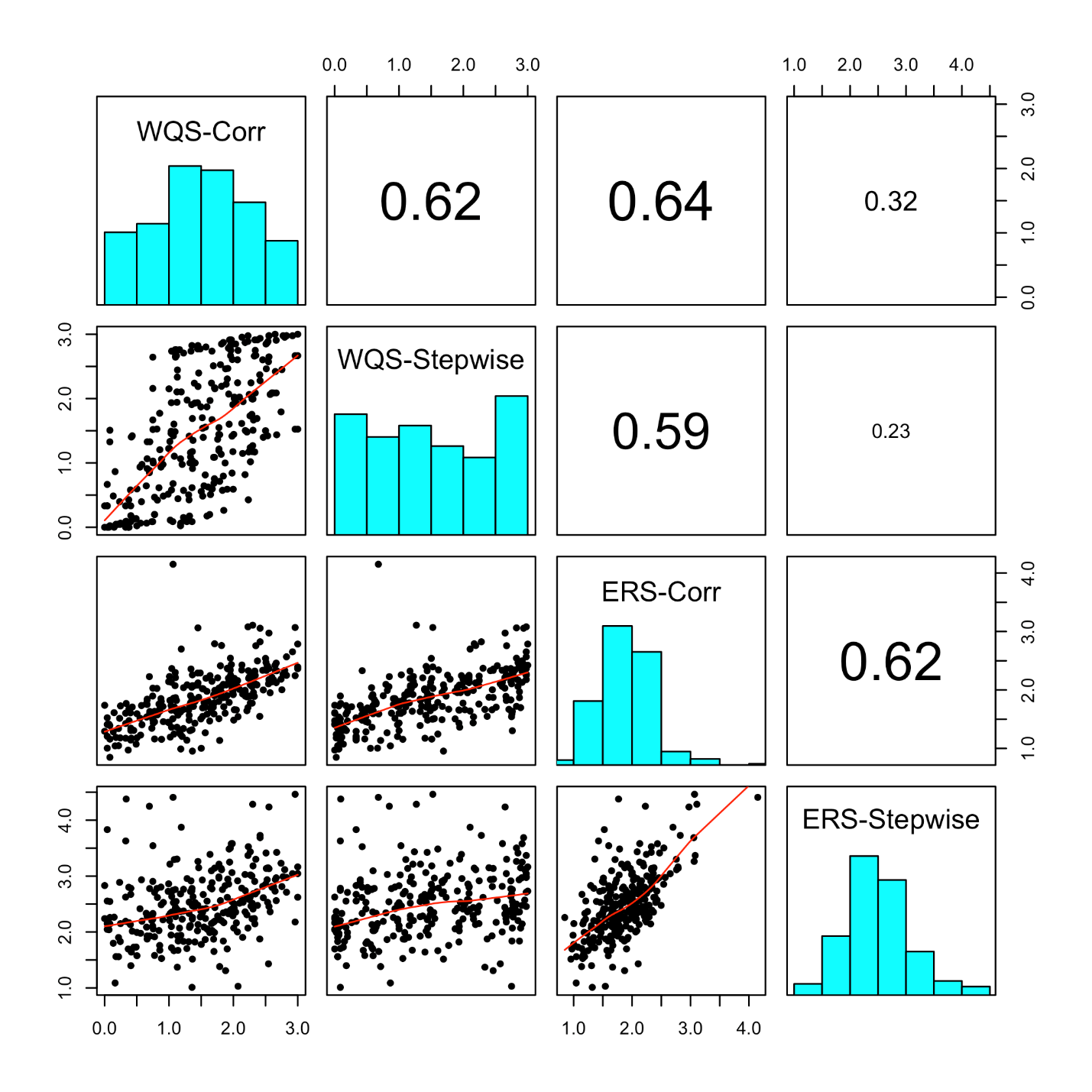
**Fig. S3** Scatterplot matrix and Pearson correlation between phthalate risk scores.

**Appendix A1: Construction of ERS and WQS in Average Exposure Analysis**

Let ${\bar{\boldsymbol{X}}}^{\boldsymbol{c}}$ denote the matrix of selected mean phthalate exposures (log-transformed) corresponding to ERS-Corr and WQS-Corr and let ${\bar{\boldsymbol{X}}}^{\boldsymbol{s}}$ denote the matrix of selected mean phthalate exposures (log-transformed) corresponding to ERS-Stepwise and WQS-Stepwise.

**ERS Construction**

To determine the weights for ERS-Corr we first fit the following logistic regression model:

$\mathrm{logit} \left( p_{i}^{c} \right)= \eta_{0}^{c}+\left( {\bar{\boldsymbol{X}}}_{\boldsymbol{i}}^{\boldsymbol{c}} \right)^{\boldsymbol{T}}\boldsymbol{\eta}_{\boldsymbol{1}}^{\boldsymbol{c}}\boldsymbol{+}{\boldsymbol{Z}_{\boldsymbol{i}}^{\boldsymbol{T}}\boldsymbol{\eta}}_{\boldsymbol{2}}^{\boldsymbol{c}}$,

where ${\bar{\boldsymbol{X}}}_{\boldsymbol{i}}^{\boldsymbol{c}}$ is the column vector of selected mean phthalate exposures for the $i$th individual, $p_{i}^{c}=P(E_{i}=1|{\bar{\boldsymbol{X}}}_{\boldsymbol{i}}^{\boldsymbol{c}},\boldsymbol{Z}_{\boldsymbol{i}})$, and $\boldsymbol{Z}_{\boldsymbol{i}}$ is a vector of covariates that includes race, education, maternal age, health insurance status, and average specific gravity over the first three visits. The estimated weights are given by ${\hat{\boldsymbol{\eta}}}_{\boldsymbol{1}}^{\boldsymbol{c}}$ and, therefore, $ERS_{i}^{c}={\bar{\boldsymbol{X}}}_{\boldsymbol{i}}^{\boldsymbol{c}}{\hat{\boldsymbol{\eta}}}_{\boldsymbol{1}}^{\boldsymbol{c}}$ for the $i$th individual.

To determine the weights for ERS-Stepwise we fit the following logistic regression model:

$\mathrm{logit} \left( p_{i}^{s} \right)= \eta_{0}^{s}+\left( {\bar{\boldsymbol{X}}}_{\boldsymbol{i}}^{\boldsymbol{s}} \right)^{\boldsymbol{T}}\boldsymbol{\eta}_{\boldsymbol{1}}^{\boldsymbol{s}}\boldsymbol{+}{\boldsymbol{Z}_{\boldsymbol{i}}^{\boldsymbol{T}}\boldsymbol{\eta}}_{\boldsymbol{2}}^{\boldsymbol{s}}$,

where ${\bar{\boldsymbol{X}}}_{\boldsymbol{i}}^{\boldsymbol{s}}$ is the column vector of selected mean phthalate exposures for the $i$th individual, $p_{i}^{s}=P(E_{i}=1|{\bar{\boldsymbol{X}}}_{\boldsymbol{i}}^{\boldsymbol{s}},\boldsymbol{Z}_{\boldsymbol{i}})$, and $\boldsymbol{Z}_{\boldsymbol{i}}$ is a vector of covariates that includes race, education, maternal age, health insurance status, and average specific gravity over the first three visits. The estimated weights are given by ${\hat{\boldsymbol{\eta}}}_{\boldsymbol{1}}^{\boldsymbol{s}}$, and, therefore, $ERS_{i}^{s}={\bar{\boldsymbol{X}}}_{\boldsymbol{i}}^{\boldsymbol{s}}{\hat{\boldsymbol{\eta}}}_{\boldsymbol{1}}^{\boldsymbol{s}}$ for the $i$th individual.

For ERS Construction, ${\bar{\boldsymbol{X}}}^{\boldsymbol{c}}$ and ${\bar{\boldsymbol{X}}}^{\boldsymbol{s}}$ are not specific gravity adjusted; specific gravity is included as a covariate in $\boldsymbol{Z}_{\boldsymbol{i}}$.

**WQS Construction**

Let $q_{ij}^{c}$ denote the quartile of the $j$th specific gravity adjusted mean phthalate metabolite in ${\bar{\boldsymbol{X}}}_{\boldsymbol{i}}^{\boldsymbol{c}}$, $j=1,\ldots,J$. To determine the weights for WQS-Corr we split the data into a 40% training set (52 Preterm Births, 140 Term Births) and a 60% testing set (78 Preterm Births, 212 Term Births) and maximize the likelihood associated with the following weighted quantile regression on 1000 bootstrapped samples (N = 192) drawn with replacement from the training data:

$logit(\theta_{i(b)}^{c})= \xi_{0}^{c}+ \xi_{1}^{c}\sum_{j=1}^{J} w_{j}^{c}q_{i(b)j}^{c}+ \boldsymbol{Z}_{\boldsymbol{i}\left( \boldsymbol{b} \right)}^{\boldsymbol{T}}\boldsymbol{\xi}_{\boldsymbol{2}}^{\boldsymbol{c}}$,

where the index $i(b)$ corresponds to the $i$th observation in the $b$th bootstrapped sample, $\theta_{i(b)}^{c}=P(E_{i\left( b \right)}=1|\boldsymbol{q}_{\boldsymbol{i}\left( \boldsymbol{b} \right)}^{\boldsymbol{c}},\boldsymbol{Z}_{\boldsymbol{i(b)}})$, and $b=1,\ldots,1000$. Specifically, $\boldsymbol{Z}_{\boldsymbol{i(b)}}$ is a vector of covariates for the $i$th observation in the $b$th bootstrapped sample which includes race, education, maternal age, and health insurance status. In maximizing the likelihood in the bootstrapped weighted quantile regressions, two constraints are imposed on the weights in the weighted quantile index, namely, $\sum_{j=1}^{J} |w_{j}^{c}|=1$ and $0\leq w_{j}^{c}\leq1$ for all $j\in\{1,\ldots,J\}$. Let $\hat{w}_{j(b)}^{c}$ denote the estimated weight for the $j$th selected mean phthalate metabolite for the $b$th bootstrapped sample of the training dataset. Then the weights for the weighted quantile sum are estimated by averaging the estimated bootstrapped weights, $\bar{w}_{j}^{c}=\sum_{b=1}^{1000} \hat{w}_{j(b)}^{c}$. The weighted quantile sum is given by:

$$WQS_{i}^{c}=\sum_{j=1}^{J} \bar{w}_{j}^{c}q_{ij}^{c}$$

Determining the weighted quantile sum for WQS-Stepwise, is identical to the weighted quantile sum construction for WQS-Corr. Let $q_{il}^{s}$ denote the quartile of the $l$th specific gravity adjusted mean phthalate metabolite in ${\bar{\boldsymbol{X}}}_{\boldsymbol{i}}^{\boldsymbol{s}}$, $l=1,\ldots,L$. To determine the weights for WQS-Stepwise we split the data into a 40% training set (52 Preterm Births, 140 Term Births) and a 60% testing set (78 Preterm Births, 212 Term Births) and maximize the likelihood associated with the following weighted quantile regression on 1000 bootstrapped samples (N = 192) drawn with replacement from the training data:

$logit(\theta_{i(b)}^{s})= \xi_{0}^{s}+ \xi_{1}^{s}\sum_{l=1}^{L} w_{l}^{s}q_{i(b)l}^{s}+ \boldsymbol{Z}_{\boldsymbol{i}\left( \boldsymbol{b} \right)}^{\boldsymbol{T}}\boldsymbol{\xi}_{\boldsymbol{2}}^{\boldsymbol{s}}$,

where the $i(b)$ index is defined above and $\theta_{i(b)}^{s}=P(E_{i\left( b \right)}=1|\boldsymbol{q}_{\boldsymbol{i}\left( \boldsymbol{b} \right)}^{\boldsymbol{s}},\boldsymbol{Z}_{\boldsymbol{i(b)}})$. Let $\hat{w}_{l(b)}^{s}$ denote the estimated weight for the $l$th selected mean phthalate metabolite for the $b$th bootstrapped sample of the training dataset. Then the weights for the weighted quantile sum are estimated by averaging the estimated bootstrapped weights, $\bar{w}_{l}^{s}=\sum_{b=1}^{1000} \hat{w}_{l(b)}^{s}$, and the weighted quantile sum is given by:

$$WQS_{i}^{s}=\sum_{l=1}^{L} \bar{w}_{l}^{s}q_{il}^{s}$$

Due to numerical instability, ${\bar{\boldsymbol{X}}}^{\boldsymbol{c}}$ and ${\bar{\boldsymbol{X}}}^{\boldsymbol{s}}$ were specific gravity adjusted prior to log-transformation in WQS Construction.

**Appendix A2: Single-pollutant models with repeated measures of exposure**

Rather than consider the average contaminant concentration for each individual phthalate, the goal of a repeated measures analysis is to account for the variation in concentrations over time in our analysis model. We will consider a two-stage method. Let $X_{ijk}$ denote the $k$th phthalate concentration for the $i$th individual measured at the $j$th visit, let $T_{ij}$ denote the gestational age for the $i$th individual at the $j$th visit, and let $SG_{ij}$ denote the specific gravity for the $i$th individual at the $j$th visit. Then stage 1 consists of fitting a linear mixed effects model with a random intercept:

$X_{ijk}=b_{0ik}+\phi_{0k}+\phi_{1k}T_{ij}+\phi_{2k}SG_{ij}+\epsilon_{ijk}$,

where the parameters $\phi_{0k}$, $\phi_{1k}$, and $\phi_{2k}$ correspond to a fixed intercept and fixed slopes across all observations for the $k$th phthalate, $k=1,\ldots,K$. $b_{0ik}$ corresponds to the random intercept for subject $i$ and phthalate $k$ and is independent of $\epsilon_{ijk}\sim N(0,\sigma_{k}^{2})$. The best linear unbiased predictor (BLUP) of the random intercept, $\hat{b}_{0ik}$, is obtained by fitting the stage 1 model and is subsequently used as a covariate in the stage 2 models after IQR normalization (denoted as $\hat{b}_{0ik}^{*}$). Stage 2 is comprised of fitting a logistic regression, a cox proportional hazards model, and an accelerated failure time model, defined as follows:

Logistic Regression:

$logit\left( \pi_{ik} \right)= \beta_{0k}+ \beta_{1k}\hat{b}_{0ik}^{*}+\boldsymbol{Z}_{\boldsymbol{i}}^{\boldsymbol{T}}\boldsymbol{\beta}_{\boldsymbol{2}\boldsymbol{k}}$, where $\pi_{ik}=P(E_{i}=1|\hat{b}_{0ik}^{*},\boldsymbol{Z}_{\boldsymbol{i}})$.

Cox proportional hazards model:

$\lambda_{k}\left( t \right)=\lambda_{0k}\left( t \right)exp(\alpha_{1k}\hat{b}_{0ik}^{*}+\boldsymbol{Z}_{\boldsymbol{i}}^{\boldsymbol{T}}\boldsymbol{\alpha}_{\boldsymbol{2}\boldsymbol{k}})$.

Accelerated failure time model:

$\log\left( T_{i} \right)=\gamma_{0k}+\gamma_{1k}\hat{b}_{0ik}^{*}+\boldsymbol{Z}_{\boldsymbol{i}}^{\boldsymbol{T}}\boldsymbol{\gamma}_{\boldsymbol{2}\boldsymbol{k}}+\sigma_{k}^{*}\epsilon_{i}$.
